# Supplementary material for: The what, why and when of adapting interventions for new contexts: A qualitative study of researchers, funders, journal editors and practitioners’ understandings
Source: PLoS One. 2021 Jul 9;16(7):e0254020. doi: 10.1371/journal.pone.0254020 (PMC8270163; doi:10.1371/journal.pone.0254020)
Supplement: S1 Appendix — (DOCX) [file pone.0254020.s001.docx]

S1 Appendix

**The Adapt Study:**

**Development of guidance for funders, researchers, policy-makers and practitioners**

**Interview Schedule: Researchers**

1. **Recording**
   1. Check with the participant that you can record the interview and switch on the recorder

*N.B. For telephone interviews it is important that the recorder is switched on before consent is taken to ensure that we have a record of consent. Ensure that they have received the paper copy of the consent form.*

1. **Consent**
   1. Ensure the participant has received and read the information sheet.
   2. Ensure the participant has the opportunity to answer any questions they have about the study.
   3. Take the participant through the consent form and explain each item. Ask the participant to initial each item and sign the form.
   4. Counter-sign the consent form.
2. **Context of Study**
   1. Introduce the study. Depending on the professional identity of the participant different levels of explanation will be required on what is adaptation.
   2. Prior to the interview select compile an overview of the intervention:
      1. Outcomes; type; target population; activities; theory of change; implementation.
      2. The context in which the intervention was originally developed/evaluated.
      3. Evaluation in the original context.
      4. The context the intervention was adapted to and/or re-evaluated in
      5. Adaptation and evaluation in the new context(s).
3. **Questions**

**Code**

- 1. Domain of question
     1. Question to ask participant
        1. *Prompts / follow-up questions*

1. Participant Details
   - 1. Can you tell me about yourself?
        1. *Current role? Previous roles? Interest in this area?*
2. What is adaptation?

Our study is considering how best to adapt or change an intervention so that in can be used in a new context, for example a different country or with a different group of people. It might include adapting intervention components, implementation or the context.

1. What does the term adaptation mean to you?
2. Why do you think intervention adaptations are undertaken?
3. What, if anything, do you think might need to be adapted for an intervention to be used in a new context? What, if anything, do you think should stay the same?
4. Are there any other terms you might use when thinking about ‘adaptation’? Can you describe them?
   1. Confirm our Knowledge of Intervention.

We are now going to talk about the intervention you were involved in adapting and / or re-evaluating in a new context (*NB. Participants might not use the term context. They might refer to country, setting etc. Use this terminology as context may seem abstract).*

*Summarise what we know about the intervention from the publications etc and confirm if this is broadly correct and if the participant has any additional information or corrections.*

1. Deciding on Adaptation
   - 1. What was your involvement with the intervention?
     2. Why was this specific intervention chosen to deliver in the new context?
        1. *Feasible, acceptable, similarity of contexts?*
     3. Was there any flexibility / adaptability built into the original intervention? If so, what was it and why? If not, why not?
        1. *What does fidelity look like in the original form of the intervention?*
     4. Can you summarise the additional changes, if any, you made to the intervention so that it could be delivered in the new context?
     5. How did you decide on what would be changed and what would stay the same?
        1. *Who decided?*
        2. *Was there consensus or disagreement? How was this resolved?*
     6. Did you undertaken any other changes that weren’t directly related to the intervention? For example did you make any changes to the setting/context? *(Explore changes beyond intervention components and discuss contextual changes if mentioned. Question will need to be amended according to how participant discusses context )*
     7. Were there any changes that you planned to undertake and did not in practice?
     8. Were there any changes that you did not plan for but happened in practice?
2. Process for Undertaking Adaptations

I understand that you undertook the following steps when adapting the intervention so that it could be delivered in the new context (outline process from study reports). Is this correct? Is there anything we have missed out?

1. How did you decide on this process?
   - - 1. *Who decided?*
       2. *Was there consensus or disagreement? How was this resolved?*
2. Was any guidance used to inform this decision-making?
   - - 1. *Which ones? How were they used? What are your reflections on them?*
3. Were there any differences between the process you intended to follow and the actual processes undertaken? If so, why?
4. Deciding on re-evaluation (*Wording and focus modified to evaluation that has been conducted)*

I understand that the intervention was re-evaluated in the new context via a pilot trial/RCT/process evaluation etc. and was found to be effective/ineffective/feasible etc. Is this correct?

1. How did you decide upon the particular approach to re-evaluation?
   - - 1. *Who decided?*
       2. *Was there consensus or disagreement? How was this resolved?*
2. Was any guidance use when deciding on the re-evaluation study design?
   - - 1. *Which ones? How were they used? What are your reflections on them?*
3. In practice were there any differences between the intended approach to re-evaluation and actual re-evaluation undertaken? If so, why?
4. How would you explain the outcome of the evaluation?
   1. *Differences in study design?*
   2. *Contexts similar/dissimilar?*
   3. *Intervention suitable/unsuitable?*
5. Overall reflection on adaptation and re-evaluation
6. What advice would you give to a researcher who was starting out with a similar study to the one you described today?
   - - 1. *What, if anything, was particularly helpful about the way you approached the adaptation and re-evaluation?*
       2. *What, if anything, was particularly unhelpful? What would you do differently in future?*
7. Reporting of adaptation
8. How was it decided how and where to report the intervention adaptation and evaluation findings?
   - - 1. *Who decided?*
       2. *Was there consensus or disagreement? How was this resolved?*
9. What influenced this decision (e.g. worked examples, guidance)?
10. Adaptation guidance
11. What are your views on having guidance to support researchers/policy-makers/practitioners in undertaking adaptation and/or re-evaluation?
12. What would useful guidance on intervention adaptation and/or re-evaluation look like to you?
13. **Closure and Dissemination**
    - Thank the participant for their time.
    - Explain what will happen with their data next (i.e. will be transferred to secure network server and anonymised)
    - Explain what will happen next in the study (i.e. DELPHI study). Ask if we can retain their details to make future contact to potentially invite them to participate in the study. Emphasise that their name will be added to the list as a relevant stakeholder in the field and not because they have participated in the qualitative study – the interviewer will anonymise the data so the participant is not known to the rest of the study team.
    - Ask if we can retain their details to make future contact in regard to dissemination (e.g. email list to circulate issued guidance)

**The Adapt Study:**

**Development of guidance for funders, researchers, policy-makers and practitioners**

**Interview Schedule: Journal Editors / Reviewers**

1. **Recording**
   1. Check with the participant that you can record the interview and switch on the recorder
2. **Consent**
   1. Ensure the participant has received and read the information sheet.
   2. Ensure the participant has the opportunity to answer any questions they have about the study.
   3. Ensure the participant has received the consent form and returned a signed copy.
   4. Counter-sign the consent form.
3. **Context of Study**
   1. Introduce the study. Depending on the journal (e.g. generic public health or specialist implementation/adaptation) different levels of explanation will be required on what is adaptation.
   2. Prior to the interview select a couple of examples of adaptation from the journal that you could discuss as concrete examples if required.
4. **Questions**

**Code**

- 1. Domain of question
     1. Question to ask participant
        1. Prompts / follow-up questions
  2. Journal and Readership
     1. Can you please tell me about your role at the journal?
     2. Can you tell me about the remit of the journal and its readership (e.g. discipline, methodological focus)?
        1. *How might its remit and readership compare with other journals within the discipline / other inter-disciplinary journals?*
        2. *Do you think studies reporting adaptations or re-evaluation is a priority for the journal? Why / why not?*
  3. Decision Making and Assessment Criteria
     1. What is the general process for making decisions about what to publish in the journal (e.g. peer review, editorial recourse)?
     2. How do you make decisions about what to publish?
        1. *Is there generic assessment criteria?*
        2. *Is there specific assessment criteria by study design etc.?*
     3. Does the journal provide reporting/publishing guidance for authors? If so, could you summarize?
  4. Interventions Reporting Adaptations and Re-evaluation
     1. If possible, could you outline any key examples of studies published in the journal that report adaptations and/or re-evaluations in new contexts?
        1. *How is adaptation defined in these studies? What do you think about these definitions?*
        2. *What are the types of interventions and outcomes presented?*
        3. *What types adaptations are presented? (Adaptation to components, implementation and/or context)*
        4. *What methodologies were presented??*
        5. *If interventions were being re-evaluated in the new context, what approaches to re-evaluation were presented? How were these justified/explained?*
     2. Does the journal set any criteria or provide guidance on how to assess either the conduct or reporting of adaptations and/or re-evaluation?
        1. *If so how are these criteria/guidance used by reviewers / how do you use them?*
        2. *What are the strengths and limitations of these criteria/guidance?*
        3. *If there is no criteria/guidance how are decisions made about whether to publish an adaptation and/or re-evaluation study? (N.B. Earlier question on general decision-making, and this is checking more specifically about adaptation studies)*
     3. *Editors:* How would you describe the nature and quality of feedback that reviewers provide for adaption and/or re-evaluation studies?
        1. *Are these any common areas of consistency and disagreement?*
     4. Based on your experience of reviewing/publishing adaptation and/or re-evaluation studies are there key strengths you have observed across studies? (e.g. tend to be a comprehensive adaptation process)
     5. Are there key limitations you have observed across studies? (e.g. poor description of rationale for adaptations)
     6. Are there key recommendations you have to strengthen studies that undertake adaptations and/or re-evaluation?
  5. Adaptation and Re-valuation Guidance
     1. Do you think guidance might support the process of deciding whether to publish adaptation and/or re-evaluation study? Why and how?
     2. What would useful guidance on intervention adaptation and/or re-evaluation look like to you?

1. **Closure and Dissemination**
   1. Thank the participant for their time.
   2. Explain what will happen with their data next (i.e. will be transferred to secure network server and anonymised)
   3. Explain what will happen next in the study (i.e. DELPHI study). Ask if we can retain their details to make future contact to potentially invite them to participate in the study. Emphasise that their name will be added to the list as a relevant stakeholder in the field and not because they have participated in the qualitative study – the interviewer will anonymise the data so the participant is not known to the rest of the study team.
   4. Ask if we can retain their details to make future contact in regard to dissemination (e.g. email list to circulate issued guidance)

**The Adapt Study:**

**Development of guidance for funders, researchers, policy-makers and practitioners**

**Interview Schedule: Funders**

1. **Recording**
   1. Check with the participant that you can record the interview and switch on the recorder

*N.B. For telephone interviews it is important that the recorder is switched on before consent is taken to ensure that we have a record of consent. Ensure that they have received the paper copy of the consent form.*

1. **Consent**
   1. Ensure the participant has received and read the information sheet.
   2. Ensure the participant has the opportunity to answer any questions he has about the study.
   3. Take the participant through the consent form and explain each item. Ask the participant to initial each item and sign the form.
   4. Counter-sign the consent form.
2. **Questions**
   1. Can you please tell me about your role on the funding panel?
   2. Funding Panel
      1. Can you tell me about the remit of the funding panel (e.g. types of study, outcome focus)?
      2. Can you tell me about the membership of the funding panel (e.g. expertise)?
      3. How do you think the funding panel’s remit and expertise fit with the wider funding context, both nationally and internationally?
   3. Decision Making and Assessment Criteria
      1. What is the process for making decisions on the panel?
         1. Prioritisation panel?
         2. Scientific panel?
      2. How do you make decisions about what to fund?
         1. Is there generic assessment criteria?
         2. Is there specific assessment criteria by study design etc.?
   4. Interventions Proposing Adaptations and Re-evaluation
      1. Does you panel have a working definition of adaptation? If so, what is it?
         1. Is there consensus/disagreement on the panel over what adaptation means?
         2. Do you use other terms to describe adaptation, and why?
      2. Does your panel set any criteria or provide guidance for applicants proposing to conduct adaptations (e.g. 6SQUiD for development)?
         1. If so how are these criteria/guidance used by applicants?
         2. How useful are these criteria/guidance to the funding panel?
      3. Can you tell me about your experience of funding studies that include proposed adaptations:
         1. What were the types, theories and outcomes of interventions proposed?
         2. What types of adaptations are proposed?
         3. What types of studies are proposed (e.g. development, outcome evaluation, and implementation)?
         4. How to studies define and analyse context?
         5. Do you have reflections on the strengths and limitations of proposed adaptions?
      4. How does the panel decide about the appropriateness of proposed adaptations?
         1. Are there key areas of consensus?
         2. Are there key areas of disagreement?
      5. How does the panel decide about the appropriateness of proposed re-evaluation?
         1. Are there key areas of consensus?
         2. Are there key areas of disagreement?
      6. Are there ways in which you think proposed adaptations and / or re-evaluation studies could be strengthened?
   5. Reporting of Adaptations in Proposals
      1. Does your panel set any criteria or provide guidance for applicants on the reporting and dissemination of adaptation studies?
         1. If so how are these criteria/guidance used by applicants?
         2. How useful are these criteria/guidance to the funding panel?
   6. Adaptation and Re-valuation Guidance
      1. Do you think guidance might support the process of deciding when to fund an adaptation and/or re-evaluation study? Why and how?
      2. What would useful guidance on intervention adaptation and/or re-evaluation look like to you?
3. **Closure and Dissemination**
   1. Thank the participant for their time.
   2. Explain what will happen with their data next (i.e. will be transferred to secure network server and anonymised)
   3. Explain what will happen next in the study (i.e. DELPHI study)
      1. Check with the study team if we would like to invite this participant to complete the DELPHI, and if so ask if they would like to be contacted about participation.
   4. Ask if we can retain their details to make future contact in regard to dissemination (e.g. email list to circulate issued guidance)
