# Supplementary material for: The what, why and when of adapting interventions for new contexts: A qualitative study of researchers, funders, journal editors and practitioners’ understandings
Source: PLoS One. 2021 Jul 9;16(7):e0254020. doi: 10.1371/journal.pone.0254020 (PMC8270163; doi:10.1371/journal.pone.0254020)
Supplement: Attachment — Submitted filename: R2R.docx [file pone.0254020.s002.docx]

| Reviewer’s comment | Response |
| --- | --- |
| Reviewer 1 |  |
| Thank you for the opportunity to review this paper. Having recent experience of adaptation, I appreciate the complexity and need to move forward this area of work. The findings and wider program of work will be of great interest to those looking to adapt public health interventions for new contexts. | We appreciate your understanding of how complex this area is and the need for clarity to help move this area forward. |
| It is unclear what data analysis (line 109) is being referred to | This has been clarified on line 109 to make it clear it is talking about the qualitative research. |
| The authors note that participants were sampled until ‘saturation’ was reached, defining this as depth and representation across the spectrum of stakeholders, yet also report that no PPI or policymakers were recruited. Does this suggest that saturation was not reached? | We have clarified that the depth and representation of stakeholders only applies to the groups of stakeholders we were able to recruit. For the groups that we did recruit we have added further clarification that we also define saturation as information power which states that the more information the sample holds the smaller the sample that is required. Our participants had a depth of knowledge and experience with adaptation that they were able to talk about. For these groups we did reach saturation. We acknowledge that we did not reach saturation for the policy makers and PPI. Please see additions in lines 199-205. |
| I was surprised that so few practitioners and policymakers participated in the interviews and wondered if this is reflective of the nature of the sampled case studies and how practitioners were involved in the adaptation process. Did the authors consider adaptations not published in the peer-reviewed literature and trial registries? | We did try to recruit policymakers, practitioners and PPI through other avenues outside of the adaptation studies. We also sampled through contacts suggested by the ADAPT study team, advertising through the Involving People charity, which aids public and patient involvement in research, and Twitter promotion targeting the European Society for Prevention Research and the Society for Prevention Research. However, these methods were not as successful as we had hoped leading to only 2 practitioners and no PPI or policymakers. This is stated in lines 166-170 in the paper. |
| On line 171 where data saturation is discussed I was unsure why this comes before recruitment of other stakeholders | The data saturation discussion has now been moved to the sample characteristic section after the section mentioned by the reviewer. Line 199-205. |
| Could the authors provide a brief summary of what key findings and evidence gaps from the systematic and scoping reviews were addressed in the topic guides? | The key gaps that were identified and addressed by the topic guide were adaptation within macro level interventions, the function of context, fidelity to the intervention functions nuances around stakeholder involvement and re-evaluation. This has been added at lines 216 to 218. |
| Did the participants also participate in the Delphi study? (line 214-215 seems to suggest this). | This has been clarified at lines 225-226 that participants were invited to take part in the Delphi study and some accepted that invitation. As the Delphi study was conducted by another study team we do not have the numbers of how many took part. |
| I was interested that participants commented on adaptation being more efficient than intervention development yet didn’t discuss the time and resources required to adapt, which can also be resource intensive. | This is discussed in further detail in our second paper which will be ready for submission to publication next month however we have added this reflection on adaption being resource intensive into the findings at line 405. The second paper is titled: Adapting population health interventions for new contexts: Understanding the experiences, practices and challenges of researchers, funders, journal editors and policy-makers. This qualitative paper examines researcher and stakeholder experiences of funding, conducting and reporting adaptation research.  We have further incorporated commentary on the time and resources in the discussion and signposted to the guidance where this has been discussed further. |
| The authors note that a 10% sample of the data was independently checked by a second researcher. Were any errors identified or amendments made following this? | We have added the results of this to line 239. The double coding led to the refinement of the definitions of some of the existing codes but did not lead to the creation of any new codes. |
| Can the authors provide a justification for using the Framework approach? | We have added justification for the framework analysis to line 229. Using this approach helped us to compare data across the cases and between the three stakeholder groups. |
| The findings were informative and well-written. Were there any differences in perspectives between the various stakeholders? | There was disagreement across the different stakeholders when it came to the definition of adaptation leading to a lack of clarity as to how this should be defined and therefore operationalised (see findings section defining adaptation). There was also disagreement between researchers and funders as to the justification of adaptation. Researchers stated that it is as time consuming as de novo development while funders stated adaptation is less resource intensive (see lines 405-414 in the findings). See lines 594 and 638 in which we have added to the discussion to show this. |
| Typos/referencing errors: lines 217, 247, 363, 455, 535, 536, 585, 612 | These have all been addressed. |
| Reviewer 2 |  |
| This is a very well written paper and covers an important and topical issue in public health globally. The authors are to be commended on their qualitative methodology which in this case has produced rich insights to guide future practice. Given the timing of this paper, the authors could also highlight the importance of this work in ensuring non communicable diseases and other risk factors are also given due focus and attention and not lost in the sole focus on Covid. Studies such as this could help countries and organisations focus their limited budgets on the breadth of public health issues by ensuring evidence from other interventions is duly translated into locally relevant practice not only to meet local needs but also reduce duplication of spend and efforts in already stretched health and social disciplines. Congratulations on this important work. | Thank you you’re your review and we appreciate your recognition of the important area this paper has addressed. We have added into the discussion a sentence to emphasize the importance of the work to public and global health work in general outside of the adaptation work during the pandemic. See line 662. |
